# Mesoscale modeling of brittle damage in polycrystalline materials with peridynamics

**DOI:** 10.1186/s41313-026-00085-5

**Published:** 2026-08-12

**Authors:** Guanfeng Zhang, Gheorghe Stan, Arun Subramaniyan, Florin Bobaru

**Affiliations:** 1 University of Nebraska-Lincoln, Lincoln, 68588 USA; 2 Material Measurement Laboratory, National Institute of Standards and Technology, Gaithersburg, 20899 USA; 3 BHGE Digital, San Ramon, USA

**Keywords:** Polycrystal, Peridynamics, Schmid factor, Fatigue, Brittle damage, Crack growth

## Abstract

A peridynamic model for polycrystalline materials is introduced to model the evolution of elastic-brittle damage under fatigue conditions. The peridynamic simulations for elastic deformations in a polycrystal are verified against corresponding Finite Element solutions for the synthetic microstructure René 88DT, an FCC polycrystalline Ni-based superalloy. The model is used to simulate the nucleation and propagation of brittle damage from fatigue loading. Damage nucleation locations obtained by the peridynamic model match those with high maximum Schmid factors for the given microstructure.

**Supplementary Information:**

The online version contains supplementary material available at 10.1186/s41313-026-00085-5.

## Introduction

Fatigue cracking is the most common cause of structural failure in aircraft components. Damage tolerant design or safety by inspection is a commonly used design method in industry, which predicts crack propagation based on fracture mechanics and periodic inspections. Understanding the crack nucleation process and incorporating microstructure information in design are still in their early stages (McDowell and Dunne 2010). Once initiated, cracks in polycrystalline materials follow complex paths, difficult to simulate with existing tools. Fatigue cracks in polycrystals can be caused by crystal plastic strain localization. In high-cycle fatigue, the applied stress is far below the yield stress. The well-known persistent slip bands (PSBs), carriers of plastic deformation, appear as the cycle number increases. The microstructural inhomogeneities produce strain localization (Miao et al. 2012). Investigating microstructure-based failure would: (1) benefit the design of crack nucleation resistant microstructures, (2) improve simulation of fatigue failure considering that the nucleation process usually takes 80 % of the total life of high cycle fatigue (Trovalusci 2016), and (3) guide material processing processes.

To describe the first stage of fatigue failure, “crack nucleation” and “crack initiation” have been used interchangeably in engineering and scientific literature. We use “crack nucleation” in this work to describe the formation of a grain level crack. It is well established that cracks in polycrystalline materials under quasistatic loads nucleate at a material surface because the grains are less constrained compared with subsurface grains, without considering the existence of precipitates, impurities, and inclusions (Suresh 1998). In terms of grain size, many experimental works show that cracks nucleate from the larger grains (Polák et al. 2010; Taira et al. 1979; Chan 2010), the reason being that larger grains enable more slipbands formation. However, recent strain-controlled fatigue experiments on solution-hardened Ni-based alloys showed that crack nucleation was controlled primarily by blocked slip transfer at grain and twin boundaries and was independent of grain size (Escobar-Moreno et al. 2025). Grains with similar crystallographic orientation form “supergrains”, which allows slip transmission across low angle grain boundaries (Chan 2010). This phenomenon was found in both Ni-based super-alloy and Ti-alloys. Miao and co-authors (Miao et al. 2012) found that in a Ni-based superalloy, most crack nucleated from grain clusters which have misorientation less than 20$^{\circ }$.

Crack nucleation near the twin grain boundaries has attracted much attention. Earlier experimental works on twin boundaries cracks are performed on copper (Boettner et al. 1964) and steels (Heinz and Neumann 1990). Recently, experiments (Miao et al. 2012) on the superalloy René 88DT at room temperature show that most cracks nucleated near the *Σ 3* twin boundaries in surface grains with size three times larger than average grain size. Experiments at elevated temperature and analytical studies (Miao et al. 2009) show that elastic incompatibility plays a key role in stress localization in twin boundaries. Highest stress concentrations are found parallel and slightly offset from the twin boundaries, and the concentration magnitude can be about 8 times the macroscopically imposed strain (Stinville et al. 2015). Based on statistical studies, Stinville et al. (2016) reported three grain-scale features that favor crack initiation of René 88DT in very high cycle fatigue: high maximum Schmid factors, long twins and elastic anisotropy. The fatigue behavior of Rene 88 DT is further explained in Stinville et al. (2018) using novel experimental techniques: the parallel slip with high elastic anisotropy are crack initiation sites for low to very high cycle regimes. Also, strain localization is caused by the annealing twins coupled with *γ '*-Ni$_3$(Al,Ti) shearing as the dominant deformation mode from intermediate to high temperature.

Besides the development of experimental techniques, advances in computational sciences make it possible to study microstructure-level analysis of polycrystalline materials. Large improvements in computational efficiency have been achieved in modeling the mechanical behavior of polycrystalline materials by using GPU-based computations, and parallel programming, and the advent of Machine Learning systems. The work in Bishop et al. (2015) studied the influence of microscale material variability on the macroscale behavior by directly simulating 420 thousand grains within an I-beam using a massively parallel finite element code. Pinz et al. (2022) combined experimental characterization, crystal-plasticity finite-element simulations, and Bayesian machine learning to develop a probabilistic model for fatigue crack nucleation in René 88DT.

Three conventional techniques of simulating crack nucleation in alloys are molecular dynamics, discrete dislocation plasticity, and crystal plasticity. Molecular dynamics simulations require a relatively small integration step size compared to the period of bond vibration and it is computationally prohibitive to model a sufficiently large number of atoms for simulating engineering-relevant problems. The advantage of the discrete dislocation plasticity method is that it can capture the formation of persistent slip bands, but 3D anisotropic models are not available for many materials, like superalloys (McDowell and Dunne 2010). Crystal plasticity is capable of capturing heterogeneous plastic deformation, texture evolution, and dislocations in metallic materials under large deformations. Coupling crystal plasticity with finite element method is the most popular approach for studying microscopic plastic deformation and potential crack nucleation. For example, Dunne et al. (2007) inserted a crystal plasticity region, with the same microstructure from measurements, into a homogenized finite element model and captured the locations of persistent shear bands and crack positions. However, the most likely predicted crack nucleation sites from this approach are not observed in experiments. Clayton (2005) combined the cohesive zone model with finite element crystal plasticity to simulate crack nucleation and propagation in a 2D plane strain microstructure model. Special treatments are needed for classical mechanics-based methods when cracks exist, with the cohesive zone model only allowing cracks to propagate along the element boundaries. More recently, crystal-plasticity phase-field formulations have been developed to reduce mesh-dependent crack-path restrictions; Liu et al. (2024) applied such a model to simulate microstructure-sensitive fatigue crack growth in a Ni-based superalloy.

Proposed by Silling (2000), peridynamics is a reformulation of the classical continuum mechanics equations that allows for a natural treatment of discontinuities in the solution by employing the concept of nonlocal interactions. Integration, rather than differentiation, is used to compute the total force density acting on a certain material volume, and deformation gradients are not used in the formulation. A distinguishing feature of the peridynamic approach, especially when combined with the so-called “meshfree discretization method” (Silling and Askari 2005), is that it allows spontaneous formation, interaction, and growth of discontinuities in a consistent framework (Xu et al. 2008). Note that PD, as a nonlocal model, can be discretized with a variety of methods, including, for example, the Finite Element Method (FEM), or various meshfree methods. For fracture problems, however, a FEM discretization is not advantageous because of its intrinsic constraints on simulating material degradation/separation. This is why the particular “meshfree method” in which the computation of the nonlocal operator is approximated by a direct evaluation of a Riemann sum or computed using the FFT (as done in, e.g., Jafarzadeh et al. (2022); Mousavi et al. (2025)) is preferred. Besides removing geometrical constraints in terms of allowing damage and material degradation to grow in autonomously and in arbitrary ways, nonlocality is also useful in modeling the evolution of damage in materials with complex microstructure (Wang and Bobaru (2026)) or under dynamic loading conditions (Wang et al. (2024)) because it allows for microscale effects to be incorporated in macroscale simulations (Xu et al. (2018); Wu et al. (2020)).

With this motivation, here we introduce a 3D mesoscale-level model of a polycrystalline material using peridynamics to simulate nucleation of cracks under fatigue loading. Peridynamic (PD) simulation of polycrystalline materials was first shown in Askari et al. (2008), in which the crack patterns along and through grain boundaries are discussed. Ghajari et al. (2014) proposed a transversely isotropic model under plane-stress and plane-strain conditions and used it to simulate dynamic brittle fracture of 2D polycrystalline microstructures. Sun and Sundararaghavan (2014) introduced a 2D cubic plasticity model based on the state-based peridynamic framework.

In the present work, the 3D bond-based PD model for polycrystalline materials with cubic symmetry grains introduced in Zhang et al. (2018) is developed to predict the stress-strain response in a three-dimensional synthetic microstructure and to observe the initiation of microcracks in the system under fatigue loading conditions. The synthetic volume element (SVE) studied in this work is representative of René 88DT microstructure of polycrystalline Ni-based superalloy, widely used for aeroengine turbines (see Krueger et al. (1992)). The peridynamic implementation is verified against a corresponding finite element model (ANSYS) in terms of strains for this representative synthetic microstructure. Strain gradients at different locations on the model are compared between the peridynamic polycrystalline model and those produced by the FE simulation to investigate if most strain concentrations are found along twin grain boundaries. Here we use a simple peridynamic “elastic-with-brittle-damage” model, to see if plasticity is needed to determine of the model captures damage nucleation sites at locations where the computed Schmid factors for the given microstructure are high. We utilize a fatigue fracture model to test, computationally, the initiation and growth of damage sites through full development of micro-cracks and cracks.

The paper is organized as follows: in Methods section, we present the PD formulation, its numerical implementation, and the PD cubic elasticity for a single-crystal. In Simulation results and validations section, we first compare the PD effective moduli with classical theoretical values and then verify the model in terms of strain variation across a grain boundary and strain concentration sites in a synthetic polycrystalline model, by comparing against FE simulations. We perform a fatigue loading test for the polycrystal sample to identify damage initiation sites and their evolution into microcracks. The correspondence between damage initiation sites obtained by the model and the locations where twin grain boundaries have high Schmid factor is discussed. Conclusions and future work are gathered in Conclusions section.

## Methods

### Brief review of Peridynamics

Peridynamics is a reformulation of the classical continuum mechanics equations that allows for a natural treatment of discontinuities in the solution by employing the concept of nonlocal interactions. The peridynamic equations of motion at a point **x** are (Silling 2000):

1 $$\begin{aligned} \rho (\textbf{x})\ddot{\textbf{u}}(\textbf{x},t) = \int _{H_\textbf{x}} \textbf{f}(\textbf{u}(\textbf{x}',t) - \textbf{u}(\textbf{x},t), \textbf{x}' - \textbf{x}) dV_{\textbf{x}'} + \textbf{b}(\textbf{x},t) \quad \text {for } \textbf{x} \in \Omega \end{aligned}$$

where *Ω* is the domain occupied by the body, $\textbf{u}$ is the displacement vector field, $\textbf{b}$ is the body force, *ρ* is the density, *t* is time, and $\textbf{f}$ is the pairwise force function in the peridynamic bond that connects material points $\textbf{x}$ and $\textbf{x}'$. The integral is defined over a neighborhood of point $\textbf{x}$, $H_\textbf{x}$, called the horizon region, or simply the “horizon”. The horizon is a sphere (disk in 2D, interval in 1D) centered at the current point with radius *δ*. Discussions on the selection and use of the peridynamic horizon and its relation to crack branching in brittle materials have appeared in Bobaru and Hu (2012); Xu et al. (2018). For a microelastic material (Silling 2000), a pairwise potential exists such that

2 $$\begin{aligned} \textbf{f}(\boldsymbol{\eta }, \boldsymbol{\xi }) = \frac{\partial \omega (\boldsymbol{\eta }, \boldsymbol{\xi })}{\partial \boldsymbol{\eta }}, \end{aligned}$$

where $\boldsymbol{\xi } = \textbf{x}' - \textbf{x}$ is the relative position vector, and $\boldsymbol{\eta }(t) = \textbf{u}(\textbf{x}',t) - \textbf{u}(\textbf{x},t)$ is the relative displacement between points $\textbf{x}$ and $\textbf{x}'$ at time *t*. A linear microelastic material is defined by a micropotential *ω* as:

3 $$\begin{aligned} \omega (\boldsymbol{\eta }, \boldsymbol{\xi }) = \frac{c(\boldsymbol{\xi })s^2\|\boldsymbol{\xi }\|}{2}, \end{aligned}$$

where the $c(\boldsymbol{\xi })$ is the micromodulus function and

4 $$\begin{aligned} s = \frac{\|\boldsymbol{\eta } + \boldsymbol{\xi }\| - \|\boldsymbol{\xi }\|}{\|\boldsymbol{\xi }\|} \end{aligned}$$

is the relative elongation of the bond connecting $\textbf{x}$ and $\textbf{x}'$. For a horizon region with spherical symmetry, the corresponding pairwise force becomes:

5 $$\begin{aligned} \textbf{f}(\boldsymbol{\eta }, \boldsymbol{\xi }) = \left\{ \begin{array}{ll} c(\boldsymbol{\xi })s \frac{\boldsymbol{\eta } + \boldsymbol{\xi }}{\|\boldsymbol{\eta } + \boldsymbol{\xi }\|}, & \|\boldsymbol{\xi }\| \le \delta \\ 0, & \|\boldsymbol{\xi }\|> \delta . \end{array}\right. \end{aligned}$$

A constructive way to build the peridynamic kernel for elasticity and convergence of the nonlocal solution to the classical solution for elastic waves propagation are discussed in Chen et al. (2016). In isotropic materials, the micromodulus function is discussed in Silling and Askari (2005) for the 3D case, in Ha and Bobaru (2010) for the 2D case, and in Bobaru et al. (2009) for the 1D case. For a 3D isotropic material, the micromodulus function is:

6 $$\begin{aligned} c(\boldsymbol{\xi }) = \frac{18k}{\pi \delta ^4} \end{aligned}$$

where *k* is the bulk modulus. For anisotropic materials, the micromodulus function $c(\boldsymbol{\xi })$ is defined by at least two parameters, and various ways to calibrate the models have been proposed (see Xu et al. (2008); Ghajari et al. (2014); Hu et al. (2011); Oterkus and Madenci (2012)). The peridynamic cubic-elasticity micromodulus (Zhang et al. 2018) is used in this work, and it will be discussed in Peridynamic cubic elasticity section.

Note that the bond-based PD introduced above cannot describe isotropic and homogeneous elastic materials with an arbitrary Poisson ratio (see Silling (2000)). The model leads to a fixed Poisson ratio of 1/4 in 3D and 2D plane strain problems, and 1/3 for 2D plane stress conditions (Trageser and Seleson (2020)). For anisotropic materials, similar limitations exist: the bond-based model can represent some specific cubic elasticities but not an arbitrary one (Zhang et al. (2018)). State-based PD models can be used for that purpose. The reason for selecting, in this work, the bond-based PD, is based on the computational efficiency of bond-based PD models when compared with other PD models (ordinary state-based, correspondence models, etc., see Mousavi et al. (2025) for a comparison of efficiency between different PD models when using the FCBM discretization approach).

In peridynamics, bonds can break irreversibly (Silling and Bobaru 2005), or reversibly (Bobaru 2007) when they are meant to represent Van der Waals-like interactions or other adhesive-type forces. For quasi-static fracture, we assume irreversibility of bond breaking, thus:

7 $$\begin{aligned} \textbf{f}(\boldsymbol{\eta }, \boldsymbol{\xi }, \textbf{x}, t) = \left\{ \begin{array}{ll} \textbf{f}(\boldsymbol{\eta }, \boldsymbol{\xi }, \textbf{x}), & \text {if } s(\boldsymbol{\xi }, \textbf{x}) < s_0 \\ 0, & \text {otherwise}, \end{array}\right. \end{aligned}$$

where $s_0$ is the critical value of bond elongation for breaking it. This critical value is defined by matching the fracture energy $G_0$ of the material to the energy required by the peridynamic model to completely separate a body in two (Ha and Bobaru 2011; Bobaru et al. 2016). In 3D, the connection between the critical relative elongation and the material fracture energy is given by Silling and Askari (2005):

8 $$\begin{aligned} G_0 = 2 \int _0^\delta \int _0^{2\pi } \int _0^{\cos ^{-1}(z/\xi )} \left( \int _z^\delta \frac{c s_0^2}{2} \xi ^2 \sin \phi \,d\phi \right) d\theta \,d\xi \,dz \end{aligned}$$

and the critical relative elongation is obtained as:

9 $$\begin{aligned} s_0 = \sqrt{\frac{5G_0}{9k\delta }} \end{aligned}$$

To monitor the evolution of damage in PD, it is useful to introduce the *damage index* quantity. In the discretized version, the nodal damage index is the ratio of broken bonds to the original number of bonds at a discretization node. This nodal quantity is usually mapped to the interval [0, 1]. A damage index of 0.5 (meaning the node has lost half of its bonds) can mean that the node is on a crack surface, if the arrangement of broken bonds is appropriate for that. In practice, given the finite-size discretization, nodes can be on the surface of a crack even if they lose only 40% of their bonds, for example (Ha and Bobaru (2011); Bobaru et al. (2016)).

### Numerical implementation of Peridynamic simulation

The peridynamic static equation is obtained by setting the acceleration term to zero in Eq. (1), and the time variable *t* represents a particular “load-step” in the deformation process:

10 $$\begin{aligned} \int _{H_x} \textbf{f}(\textbf{u}(\textbf{x}',t) - \textbf{u}(\textbf{x},t), \textbf{x}' - \textbf{x}) dV_{\textbf{x}'} + \textbf{b}(\textbf{x},t) = 0 \quad \text {for } \textbf{x} \in \Omega \end{aligned}$$

The region defining the material is discretized with a uniform grid with spacing *Δ x* and nodes centered in the middle of their corresponding volume (cuboid) produced by the discretization. We use a one-point Gaussian quadrature (mid-point integration) over the domain to approximate the value of the integral. The discretized version of Eq. (10) is:

11 $$\begin{aligned} \sum \limits _{j \in H_i} \textbf{f}(\textbf{u}_j, \textbf{u}_i, t^n) V_{ij} + \textbf{b}_i^n = 0 \end{aligned}$$

in which *i*, and *j* are node numbers, $V_{ij}$ is the portion of the volume of node *j* covered by the horizon of node *i*, and superscript *n* is the load-step number.

The peridynamic bond force in Eq. (5) is linear in displacements in the 1D case (see Bobaru et al. (2009)). In 2D and 3D cases, however, the relation is nonlinear in general, and is linear only if the relative displacement vector, $\textbf{u}(\hat{\textbf{x}}, t) - \textbf{u}(\textbf{x}, t)$, is aligned with the bond $\boldsymbol{\xi }$ direction, which happens only in very special deformation cases. The expression of the bond force in Eq. (5) in terms of relative displacements is:

12 $$\begin{aligned} \textbf{f}(\boldsymbol{\xi }, \textbf{u}, \textbf{x}, t) = \left\{ \begin{array}{ll} \dfrac{\textbf{u}(\hat{\textbf{x}}, t) - \textbf{u}(\textbf{x}, t) + \boldsymbol{\xi }}{\left\| \textbf{u}(\hat{\textbf{x}}, t) - \textbf{u}(\textbf{x}, t) + \boldsymbol{\xi } \right\| } \, c(\boldsymbol{\xi }) \dfrac{\left\| \textbf{u}(\hat{\textbf{x}}, t) - \textbf{u}(\textbf{x}, t) + \boldsymbol{\xi } \right\| - \Vert \boldsymbol{\xi }\Vert }{\Vert \boldsymbol{\xi }\Vert }, & \text {if } s(\boldsymbol{\xi }, \textbf{x}) < s_0, \\ 0, & \text {otherwise}. \end{array}\right. \end{aligned}$$

Solving the nonlinear algebraic system (in nodal displacements) in Eq. (11) requires the use of a nonlinear system solver. From the principle of minimum potential energy, solving the nonlinear system (in Eq. (11)) is equivalent to minimizing the potential energy. The Euler–Lagrange equations for the system are obtained by finding the gradient of the potential energy with respect to displacements. Note that in PD, traction boundary conditions are implemented as body forces. Therefore, one can obtain the solution to the static PD elasticity problem by the energy minimization method (Le (2014)). The Nonlinear Conjugate Gradient (NCG) method (Fletcher and Reeves 1964) is adopted here.

The potential energy, $\Pi = W - W_{\textrm{ext}}$, is the difference between the internal strain energy in the system, *W*, and the work done by external forces, $W_{\textrm{ext}}$. The internal strain energy is:

13 $$\begin{aligned} W = \int _{\Omega } \omega \, dV, \end{aligned}$$

in which the strain energy density *ω* for each node is defined by Eq. (3). The work done by the external forces is:

14 $$\begin{aligned} W_{\textrm{ext}} = \int _{\Omega } \textbf{p}_{\textbf{x}} \, \textbf{u}_{\textbf{x}} \, dV, \end{aligned}$$

where $\textbf{p}_{\textbf{x}}$ is the force density vector on node $\textbf{x}$. This term includes the tractions applied on the boundary of the system, which, in the nonlocal model are applied on a thin volume (in 3D) along the corresponding surface.

For the NCG, we use the Fletcher–Reeves formula (Fletcher and Reeves 1964) with secant method for the line search. Possible stopping criteria for the NCG iterations, at iteration *k*, are:

- $$\Pi _k = (W - W_{\textrm{ext}})_k < \textrm{tol},$$
- $$|W_k - W_{k-1}| < \textrm{tol},$$
- $\Vert \textbf{F}_k\Vert _2 < \textrm{tol},$ for norm-2 of the total force vector.

In this work we chose the second criterion, with tol=$10^{-6}$.

### Peridynamic cubic elasticity

The material microstructure used in this work is René 88DT. Due to the cubic symmetry of its grains, René 88DT grains have three independent elastic constants: Young’s moduli $E_1=E_2=E_3= 97.62$ GPa, Poisson ratios $v_{12}=v_{23}=v_{31}= 0.41$, and shear moduli $G_{12}=G_{23}=G_{31}=108.00$ GPa.

In the bond-based PD model for cubic elasticity, the PD micromodulus surface is described by Zhang et al. (2018):

15 $$\begin{aligned} \frac{1}{c(\alpha ,\beta ,\gamma )} = \frac{A_1}{E} \left(\alpha ^4+\beta ^4+\gamma ^4\right) + A_2 \left( \frac{-2v}{E} + \frac{1}{G} \right) \left(\beta ^2\gamma ^2+\gamma ^2\alpha ^2+\alpha ^2\beta ^2\right), \end{aligned}$$

where parameters $\alpha , \beta , \gamma$ are the directional cosines of the bond direction (the cosines of the angles between the bond direction and the material coordinates axes). Two constants $A_1$ and $A_2$ are to be determined. By enforcing a match between longitudinal wave propagation speeds along three particular directions, least squares minimization was used in Zhang et al. (2018) to calculate these two parameters. In this work, we use a similar approach to obtain $A_1$ and $A_2$, but instead of matching the wave propagation speeds like in the dynamic case, since we solve a static problem, we enforce matching the effective moduli along three directions ({100}, {110}, and {111}) in the single crystal. This procedure is described in the next section.

It is important to note that the directional micromodulus formula in Eq. (15) for cubic crystals is derived from the general orthotropic compliance expression by applying cubic symmetry, which enforces the equivalence of all three crystallographic axes and reduces nine independent elastic constants to three, in the classical model. This symmetry is shared by all cubic crystals (BCC, FCC, etc.) regardless of their specific atomic arrangement. Therefore, the calibrated parameters for the peridynamic micromodulus surface retain the same physical meaning across all cubic materials, while their numerical values differ from one material to another. The calibrated values from static problems can be used in other types of analysis, if the material response remains within the linear elastic regime. The calibrated values of $A_1$ and $A_2$ in the current study differ from those reported in Zhang et al. (2018), and this is expected because the two studies model different materials with different elastic constants.

## Simulation results and validations

### Orientation-dependence of elastic modulus in a cubic crystal

In this section, we show that the 3D bond-based peridynamic model discussed above for cubic crystals is able to capture the effective modulus along different directions of a single grain. A cubic single-crystal sample (20 *μ*m *×* 20 *μ*m *×* 20 *μ*m) with three different orientations (given by the Euler angles $(0^\circ , 0^\circ , 0^\circ )$ in the ZXZ notation, $(0^\circ , 45^\circ , 0^\circ )$, and $(45^\circ , 55^\circ , 0^\circ )$) is considered. Three different horizon sizes (*δ*) are used: 3 *μ*m, 2 *μ*m, and 1.6 *μ*m for each case, with uniform discretizations. The spacing between the discretization nodes, *Δ x*, is so that the horizon factor $m = \frac{\delta }{\Delta x} = 4$, for all simulations. This means that when a smaller horizon is used, the grid density is increased so that the horizon factor stays the same (see Bobaru et al. (2009) for *δ*-convergence). Samples are loaded in tension by applying a force density, equivalent to a stress value of *σ = 500* MPa, on the top surface nodes and fixing the translational degree of freedoms on the bottom surface nodes only in the direction of the applied load. We solve the quasi-static deformation problem, using the micromodulus defined by Eq. (15), and find displacements at all nodes in the sample. To estimate the effective modulus in the loading direction for the sample with a specific crystalline orientation, we first calculate the strain *ε*, in the loading direction, using the discrete central difference for the displacements in the loading direction, *u*, of peridynamic nodes along the center line of the sample except for the nodes near (within 2*δ* from) the top and bottom surfaces (to avoid the well-known surface effect, see Le and Bobaru (2018)). Then, we compute the effective modulus as $E_\textrm{eff} = \frac{\sigma }{\epsilon }$.

**Table 1:** Effective moduli for a cubic crystal calculated by the cubic elasticity PD model for different horizon sizes (and corresponding grids to maintain the horizon factor value of *m=4*). Relative differences from the classical theoretical values are shown in parentheses

Directions	Euler angles	Analytical	Effective modulus (GPa)		
	(ZXZ conv.)	(GPa)	PD $\delta =3.0 \mu$m	PD $\delta =2.0 \mu$m	PD $\delta =1.6 \mu$m
{100}	(0, 0, 0)	97.6	99.0 (1.43%)	102.2 (4.71%)	95.0 (2.66%)
{110}	(0, 45, 0)	188.3	183.0 (2.81%)	186.4 (1.01%)	185.2 (1.65%)
{111}	(0, 55, 45)	271.8	286.7 (5.48%)	277.8 (2.21%)	272.2 (0.15%)

Table 1 compares the peridynamic effective moduli computed for the loading direction for each of the three crystallographic orientations with the corresponding classical analytical values. Uniform discretizations with a horizon factor *m = 4*, produced results that matched well experimental observations in the edge-on impact test, when the horizon size was sufficiently small relative to the grain size, as reported in Zhang et al. (2018). For the quasistatic loading discussed here, differences of effective moduli between the peridynamic simulations and analytical values from the classical theory decrease, in general, as the horizon size *δ* decreases from 3.0 *μ*m to 1.6 *μ*m, but note that they are not expected to necessarily reach zero in the limit of the horizon going to zero (*δ*-convergence, as defined in Bobaru et al. (2009)) because of the limitations of the bond-based PD model in terms of the Poisson ratio mentioned in Brief review of Peridynamics section. For cubic elasticity, this model may match a select few cubic models, but only approximate the rest.

### Strain variations across a grain boundary

In this section we compare strains between the PD model (discretized using the meshfree method) and classical elasticity (discretized using the FEM) across a grain boundary. One could compute the nonlocal strains in the PD model, but here, instead, we use the displacements found with the PD model to compute strains based on finite differences approximations, and compare them with those obtained from the FE solution. Note that in the PD model (all except PD correspondence models), strains do not appear directly in the formulation, and all calculations are based on relative displacements. Thus, from the PD results for displacements, we compute approximate nodal strains as a post-processing step to compare with those provided by the FE analysis.

Note that grain boundary (GB) properties are affected by a number of factors, such as inclusions, other phases, pores, etc. In the absence of specific knowledge about grain boundary properties, here we select a particular type of averaging for PD bonds that cross a grain boundary. We will compare the PD results with those produced from the FEM model in which the elastic properties of the GB do not explicitly enter the picture.

The microstructure (grain orientation, grain boundaries, etc.) plays a critical role in the evolution of damage in a polycrystalline alloy. Representing the material interfaces (grain boundaries) in such systems is a key component in simulating crystalline materials deformation. A simple algorithm for bond properties was discussed in the peridynamic simulation of a polycrystal (Zhang et al. 2018), where the smaller value of the micromodulus calculated from two sets of material properties was adopted if a bond crosses two grains. In this section, we describe a different way of assigning peridynamic parameters for bonds that cross a grain (or twin) boundary and we test the convergence of the peridynamic results to the classical result in terms of normal strain across a material interface.

**Fig. 1:**
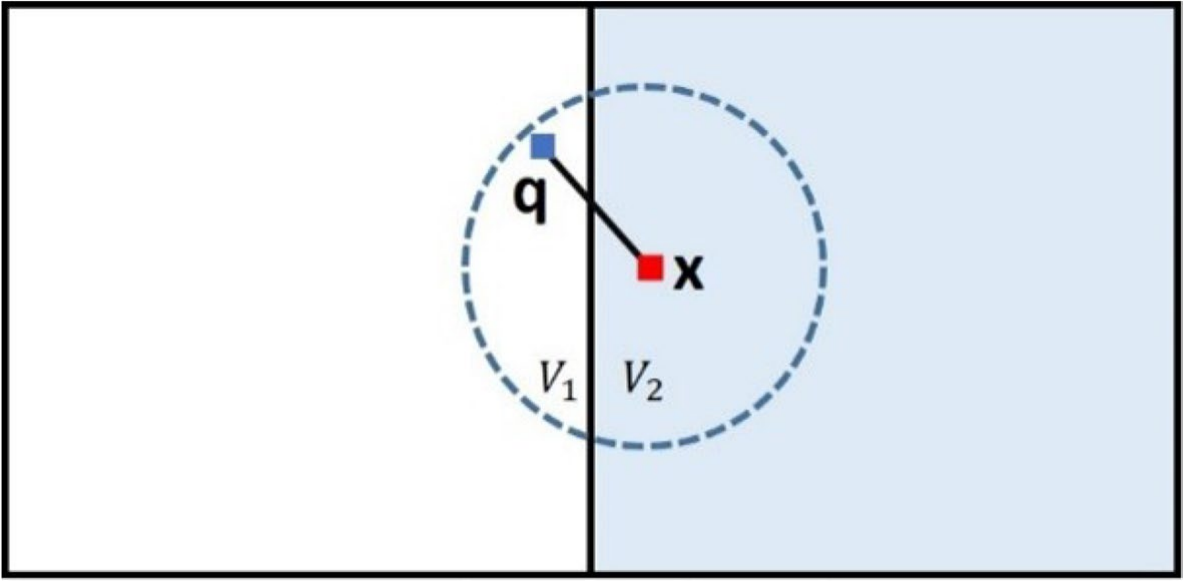
When the horizon of point $\textbf{x}$ covers two material phases, the bond $\boldsymbol{\xi } (\textbf{x},\textbf{q})$ crossing a material interface has properties computed using Eq. (16)

Similar to other nonlocal methods, simulating a material interface is different from local models because a peridynamic bond links, across the interface, two material points with different material properties (see bond $\boldsymbol{\xi } (\textbf{x},\textbf{q})$ connecting material points $\textbf{x}$ and $\textbf{q}$ in Fig. 1).

To calculate the micromodulus of a bond crossing the interface, averaged material properties are used. The effective material properties at a point **x** are calculated based on the volume percentage of two materials covered by its horizon region:

16 $$\begin{aligned} E(\textbf{x}) = \frac{E_1 V_1 + E_2 V_2}{V_1 + V_2} \end{aligned}$$

where $E_1$ and $E_2$ are Young’s moduli of the two material phases and $V_1$ and $V_2$ are the corresponding volumes (or areas for the 2D case) of these two phases covered by the horizon of $\textbf{x}$ (see Fig. 1). Other material properties are calculated in a similar way. The volume-weighted formula in Eq. (16) is justified because it leads to a smoothing of properties as one crosses from one grain to another.

**Fig. 2:**
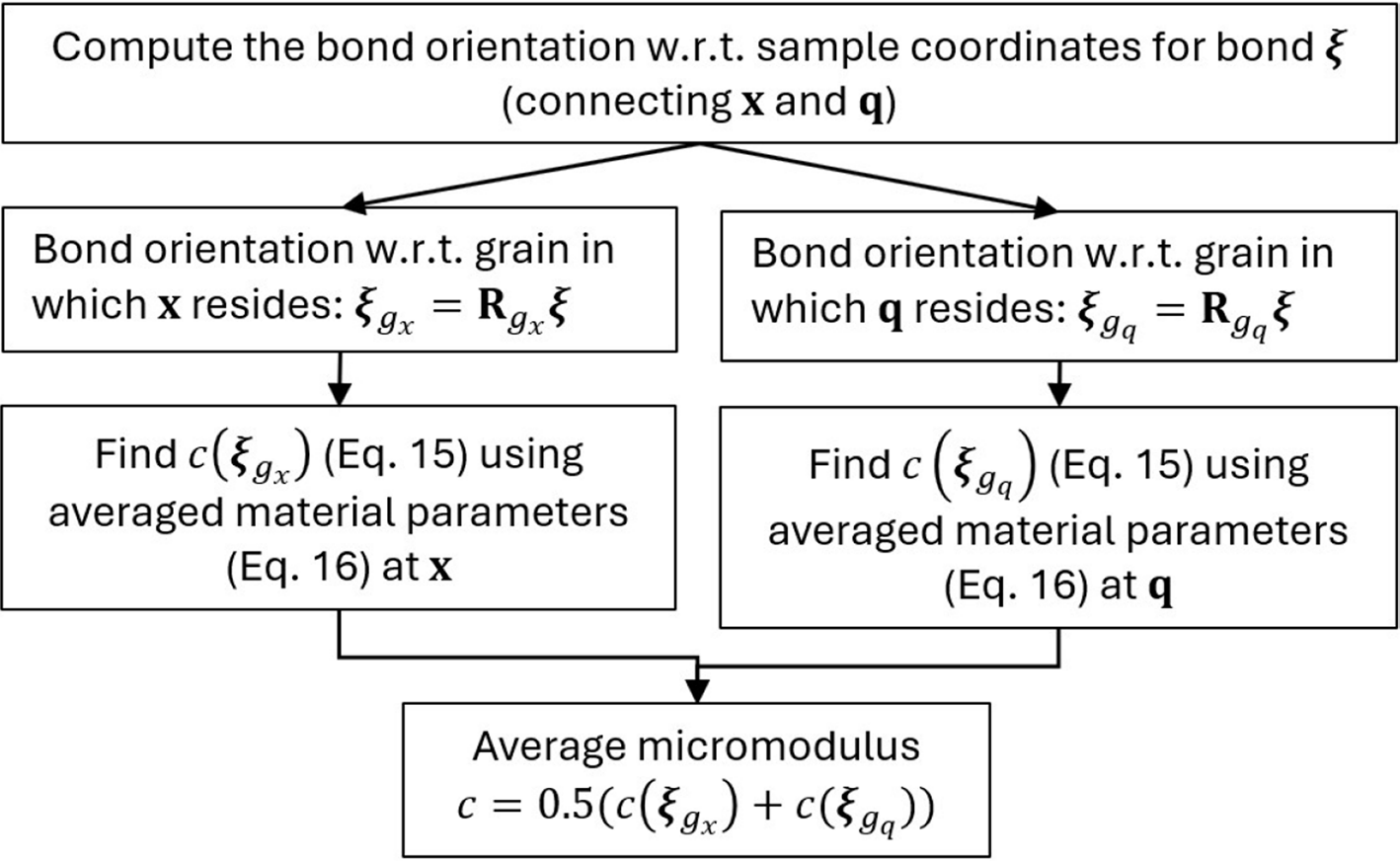
Flowchart for calculating the micromodulus of a peridynamic bond that crosses a grain boundary in a polycrystalline material. The bond $\boldsymbol{\xi }(\textbf{x},\textbf{q})$ connects material points $\textbf{x}$ and $\textbf{q}$, which reside in grains $g_x$ and $g_q$, respectively. Matrices $\textbf{R}_{g_x}$ and $\textbf{R}_{g_q}$ are the rotation matrices for grains $g_x$ and $g_q$, as defined by Eq. (17). The transformed vectors $\boldsymbol{\xi }_{\textbf{g}_\textbf{x}}=\textbf{R}_{g_x}\boldsymbol{\xi }$ and $\boldsymbol{\xi }_{\textbf{g}_\textbf{q}}=\textbf{R}_{g_q}\boldsymbol{\xi }$ represent the bond orientation in the coordinate systems of grains $g_x$ and $g_q$, respectively

For a bond with its ends in the same grain, we calculate the micromodulus of the bond using the single-crystal PD model Eq. (15). When a bond $\boldsymbol{\xi }(\textbf{x},\textbf{q})$ connects material points within two different grains (see Fig. 1), the micromodulus calculation is shown in the flowchart given in Fig. 2. For such bonds, we compute the bond orientation with respect to the sample coordinates and then with respect to the grains in which $\textbf{x}$ and $\textbf{q}$ reside, respectively. This allows us to compute two intermediate bond micromoduli, using Eq. (15) for each of the grains. The “grain” elastic parameters needed in Eq. (15) are computed using the volume-average idea in Eq. (16). The final value of the micromodulus for a bond crossing a grain boundary is the average of the two intermediate bond micromoduli.

As illustrated in Fig. 2, the bond orientation is transformed using a rotation matrix $\textbf{R}$. Following the ZXZ convention for Euler angles $(\psi _1,\Theta ,\psi _2)$, the matrix $\textbf{R}$ is defined as (see, e.g., Diebel (2006)):

17 $$\begin{aligned} \textbf{R} =\left[ \begin{array}{ccc} \cos \psi _1\cos \psi _2-\sin \psi _1\cos \Theta \sin \psi _2 & -\cos \psi _1\sin \psi _2-\sin \psi _1\cos \Theta \cos \psi _2 & \sin \psi _1\sin \Theta \\ \sin \psi _1\cos \psi _2+\cos \psi _1\cos \Theta \sin \psi _2 & -\sin \psi _1\sin \psi _2+\cos \psi _1\cos \Theta \cos \psi _2 & -\cos \psi _1\sin \Theta \\ \sin \Theta \sin \psi _2 & \sin \Theta \cos \psi _2 & \cos \Theta \end{array}\right] . \end{aligned}$$

**Fig. 3:**
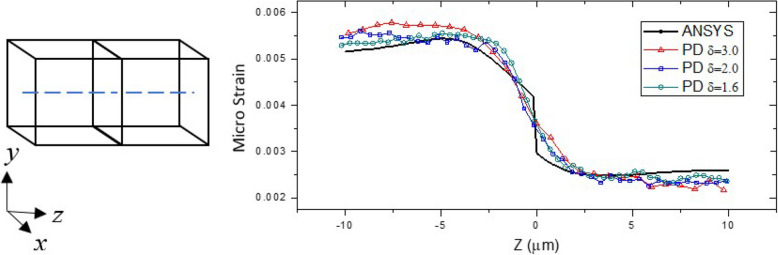
A sample with two grains (left) that have different orientations is used to study the strain variation across an interface. Subject to tensile loading along the *z*-direction, the figure on the right shows the normal strain values along the center line obtained with ANSYS and the PD simulations with several horizon sizes

A 3D sample with two cubic grains along the *z*-axis is simulated to validate the peridynamic simulation with grain boundaries. Geometry of each grain is 10 *μ*m *×* 10 *μ*m *×* 10 *μ*m (see Fig. 3). The discretization for this peridynamic model has a uniform grid (as discussed above), and three different horizon sizes are used: 3 *μ*m, 2 *μ*m, and 1.6 *μ*m. The sample is loaded in tension by fixing the translational degrees of freedom for nodes on the right surface and applying 0.5 MPa tensile stress on nodes on the left surface. The orientation of these two grains is (0, 0, 0), and (0, 45, 0), respectively, which produce effective moduli along the loading direction of 97.6 GPa and 188.3 GPa, respectively. Figure 3 shows the normal strain variation across the interface between the two grains.

The ANSYS FEM-based simulation of the corresponding classical elasticity model was performed. The reference model was discretized using 2,000 SOLID186 quadratic elements (9581 nodes) with a uniform 1 *μ*m element edge length. Using a finer mesh did not change the solution in a significant way. Crystallographic orientation was defined via local Element Coordinate Systems (ESYS): the left grain was aligned with the global Cartesian basis, while the right grain was rotated 45$^\circ$ about the *x*-axis.

Note that the strain values in PD and FE are computed in different ways. In the PD simulations, the normal strain at a material point is approximated by a central difference of displacement values of adjacent points and forward/backward difference for boundary points along the desired direction. Nodal strains in the FE simulations are extrapolated from the integration points in the element. As expected, the grain with smaller effective modulus has larger strain. Near the grain boundary, we can see an abrupt strain variation. Different from the sharp (discontinuous) drop of strain from ANSYS, peridynamic simulations give a smoother transition for strain across the interface between grains. As the horizon size decreases, we find the match between the peridynamic simulations and ANSYS results improves. Also, the strain variation across the interface gets sharper with the smaller horizon size. The thickness of the region where strains are mollified in the PD results compared with the jump-discontinuity of the ANSYS result, is about 2*δ*, because of the *δ* thickness of the region at a free surface known as the “PD surface effect” or “PD skin effect” (Le and Bobaru (2018)). The peridynamic surface effect results from the fact that the calibration of the parameters in the peridynamic model assumes points to be in the bulk, thus having their full horizon region inside the body. Near a surface, however, the horizon region is not complete. This material region (*δ* from the free surface) behaves effectively softer than the material in the bulk (see Le and Bobaru (2018)).

This analysis leads us to conclude that in order to capture the high gradients in strains at a grain boundary, a PD model needs to use a sufficiently small horizon size relative to the grain size. We also emphasize that the ANSYS solution is not necessarily the “ground truth” solution, for the following reason: grain boundaries are never perfectly straight mathematical lines (because of impurities, vacancies, misorientation, etc.), and, as a consequence, one should not expect a mathematical sharp discontinuity in strains across a real grain boundary. From this perspective, the nonlocal smoothing provided by the PD solution is an advantage.

**Remark:** the nonlocal interactions in peridynamics do not refer to the direct physical interactions between atoms, even though this is possible when the simulation scale is atomistic and the force in Eq. (1) is the atomic interaction force. For dynamic problems, the peridynamic horizon *δ* may be viewed as an effective length scale of a continuum model. This length scale can sometimes be suggested by experimental results (Ramulu et al. (1983); Xu et al. (2018)). For example, to reproduce certain dynamic brittle failure features, the horizon size should be smaller than the damage thickness for isotropic materials, and smaller than the grain size for polycrystalline materials (see Zhang et al. (2018)).

### Strain concentrations sites in René 88DT microstructure

Damage and crack nucleation occur at strain concentration sites. We study the strain concentration sites in a synthetic polycrystalline model. PD results are compared with those of FE simulations performed with ANSYS.

An important remark needs to be made: in the PD simulations we use uniform grids, easy to generate, while the FE simulations in this work use conforming meshes, which conform to the grain and twin boundaries geometry of the microstructure. A conforming mesh is more costly to build than a non-conforming, uniform mesh but provides more accurate description of the boundaries/interfaces. The differences between FE solutions for elastic and elastoplastic behavior of polycrystalline materials using conforming and non-conforming meshes are studied in Pyle et al. (2013). Those results show that uniform mesh is more computationally efficient and the two discretization types provide almost identical macroscopic stress-strain behavior, statistical distribution of stress, and overall texture evolution (Pyle et al. (2013)). However, especially around grain boundaries and interfaces, differences between the geometry-conforming and the uniform mesh are noted.

**Fig. 4:**
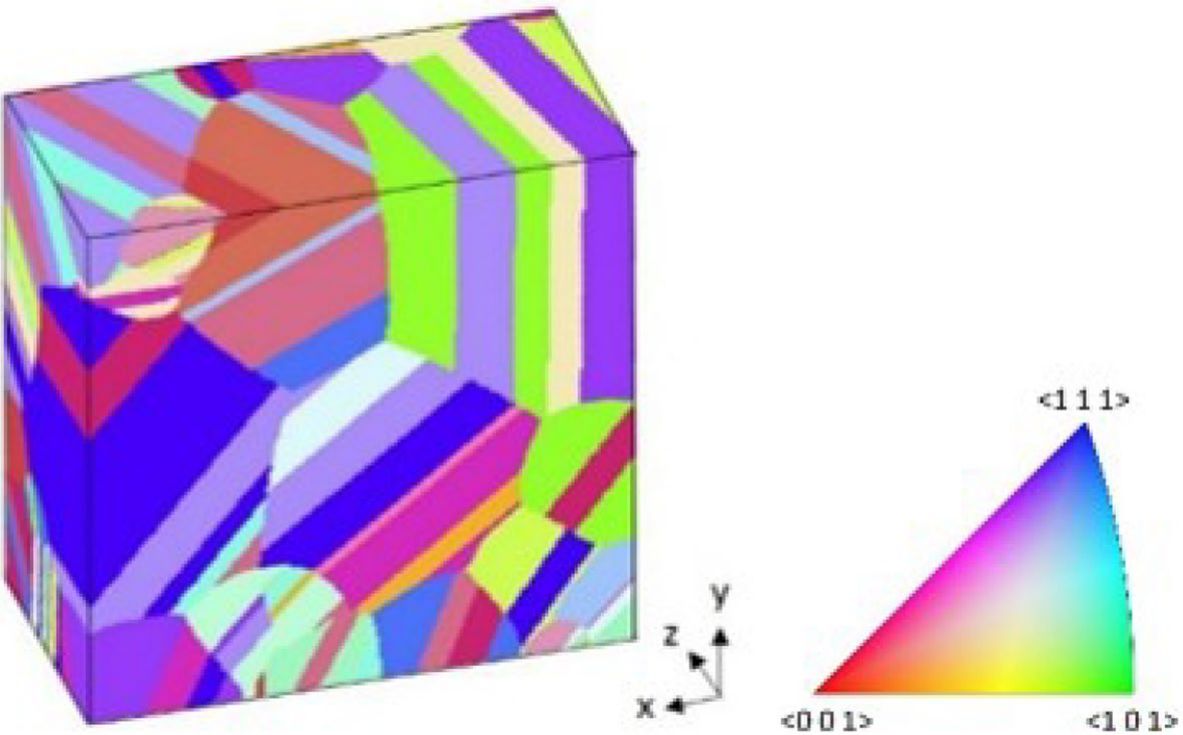
Microstructure of the René 88DT synthetic model (with 179 grains) and the inverse pole figure color key

We consider the following setup for analyzing the strain concentration sites in polycrystalline materials: a synthetic René 88DT model with dimensions of 100 *μ*m *×* 100 *μ*m *×* 50 *μ*m as shown in Fig. 4. This model has 179 grains with known orientation and most of them are twin grains. This microstructure is generated by DREAM3D (Groeber and Jackson (2014)) based on statistical data from experimental measurements. Voxel data from DREAM3D is then used to build a peridynamic model. The peridynamic model uses uniform meshes ($\Delta x=\Delta y=\Delta z$). Three peridynamic simulations are performed using different horizon sizes (and corresponding grid densities), see parameters listed in Table 2. Nodal coordinates in the discretized model start from the origin to 100 *μ*m, 100 *μ*m, 50 *μ*m in 3D. The sample is in tension by applying a force density equivalent to 500 MPa tensile stress on the top nodes (*y = 100* *μ*m) and fixing the translational degrees of freedom (in the loading direction only) of nodes on the bottom surface (*y = 0* *μ*m).

**Table 2:** Peridynamic parameters for computations with different horizon sizes

Horizon (*μ*m)	Node spacing (*μ*m)	# nodes	Comp. cost (hours)
3.0	0.75	1,167,474	5.8
2.0	0.50	4,000,000	11.7
1.6	0.40	7,812,500	24.6

**Fig. 5:**
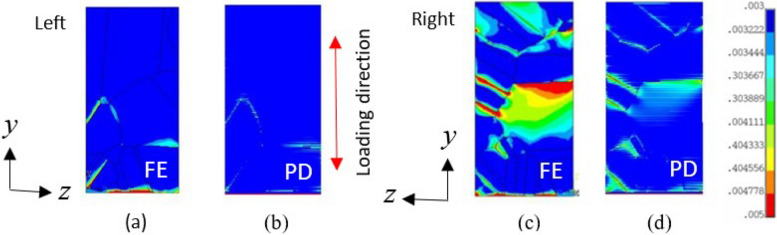
Comparison of normal strain $\varepsilon _y$ on the left (in **a** and **b**) and right (in **c** and **d**) surfaces of the sample between FE (in **a** and **c**) and PD (in **b** and **d**) simulation results

**Fig. 6:**
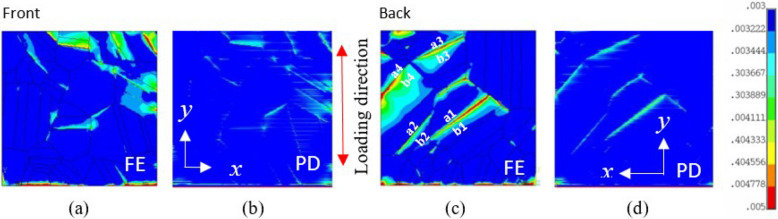
Comparison of normal strain $\varepsilon _y$ on the front (in **a** and **b**) and back (in **c** and **d**) surfaces of the sample (see Fig. 4) between the FE (in **a** and **c**) and PD (in **b** and **d**) simulation results. All strain plots use the same legend for the color scheme

**Fig. 7:**
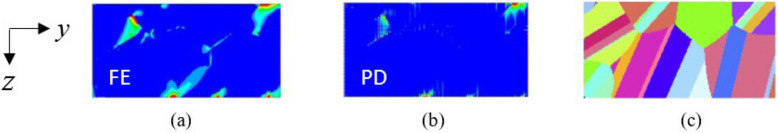
Comparison of normal strains $\epsilon _y$ from the FE (in **a**) and PD (in **b**) simulation results for the middle plane-cut *x=50* *μ*m, and its corresponding microstructure (in **c**)

**Fig. 8:**
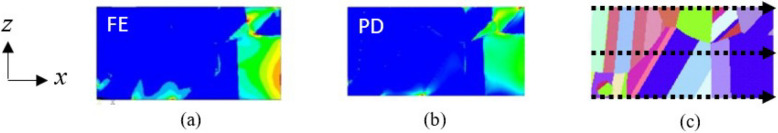
Comparison of normal strains $\epsilon _y$ from the FE (in **a**) and PD (in **b**) simulation results for the middle plane-cut *y=50* *μ*m, and its corresponding microstructure (in **c**). The dotted lines in (**c**) are for the extraction of strain data shown in Figs. 11, 12, 13 and 14

**Fig. 9:**
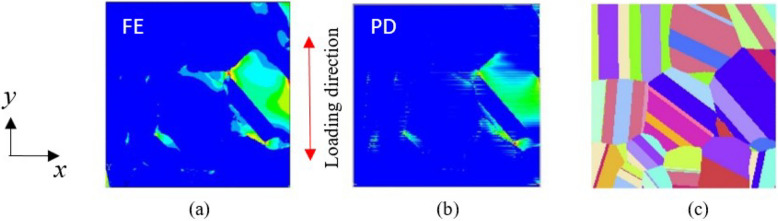
Comparison of normal strains $\epsilon _y$ from the FE (in **a**) and PD (in **b**) simulation results for the middle plane-cut *z=25* *μ*m, and its corresponding microstructure (in **c**)

**Fig. 10:**
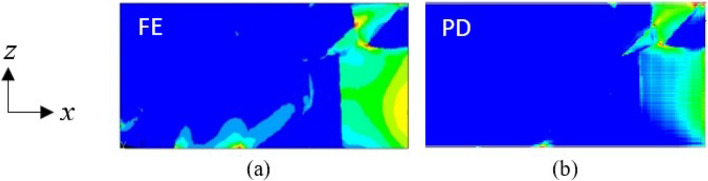
Comparison for $\epsilon _\textrm{eq}$ between FE (**a**) and PD (**b**) over the middle plane (*y=50* *μ*m), the same as used in Fig. 8

**Fig. 11:**
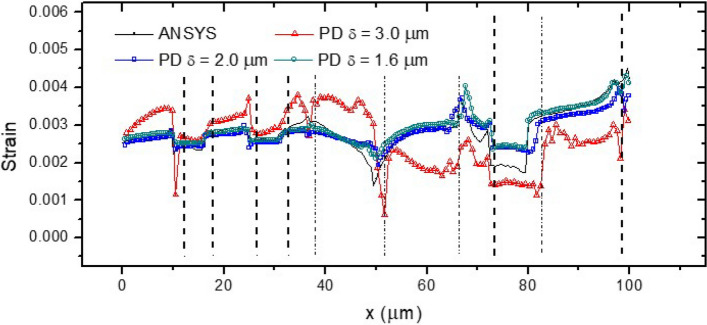
Comparison for $\epsilon _y$ between ANSYS and three PD simulations with different horizon sizes, along the intersection of *y = 50* *μ*m and *z = 50* *μ*m planes (top line in Fig. 8c). Vertical dashed lines and dash-dot lines indicate locations of twin boundaries and grain boundaries along this line, respectively

**Fig. 12:**
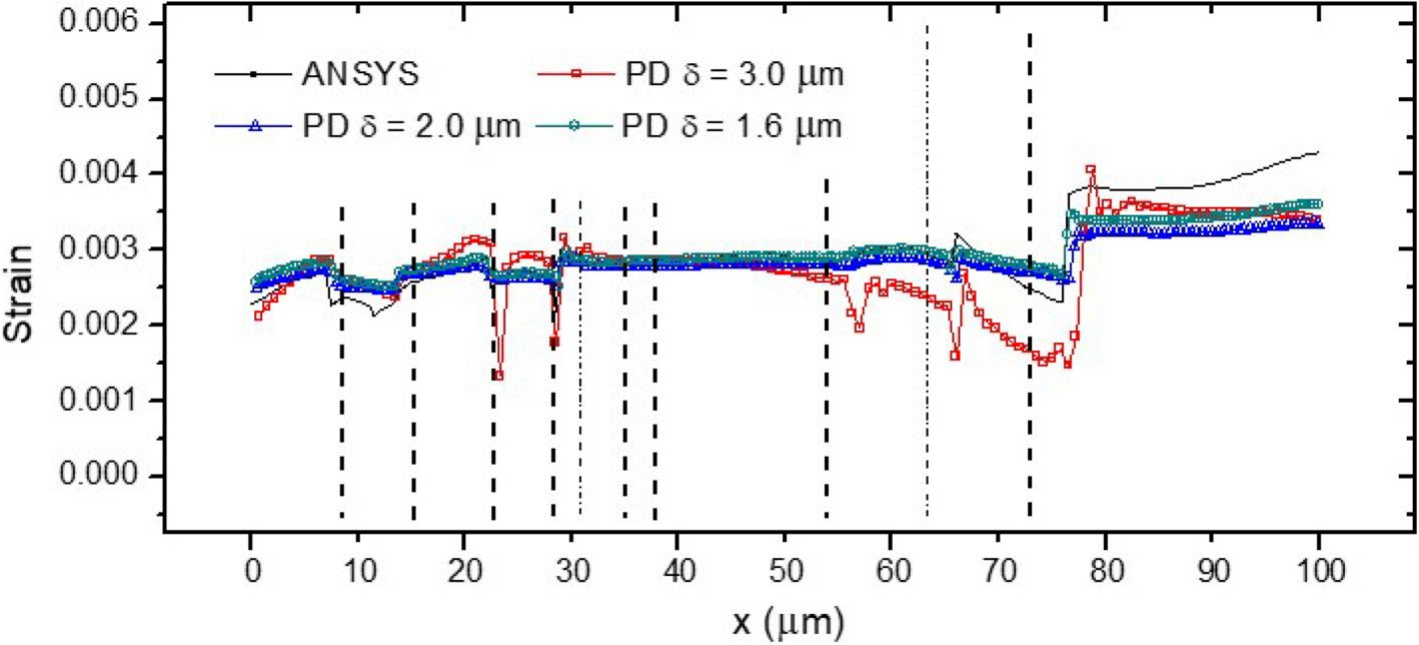
Comparison for $\epsilon _y$ between ANSYS and three PD simulations with different horizon sizes, along the intersection of *y = 50* *μ*m and *z = 25* *μ*m planes (center line in Fig. 8c). Vertical dashed lines and dash-dot lines indicate locations of twin boundaries and grain boundaries along this line, respectively

**Fig. 13:**
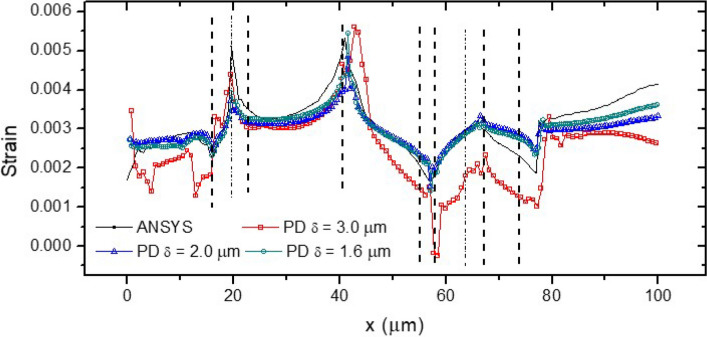
Comparison for $\epsilon _y$ between ANSYS and three PD simulations with different horizon sizes, along the intersection of *y = 50* *μ*m and *z = 0* *μ*m planes (center line in Fig. 8c). Vertical dashed lines and dash-dot lines indicate locations of twin boundaries and grain boundaries along this line, respectively

**Fig. 14:**
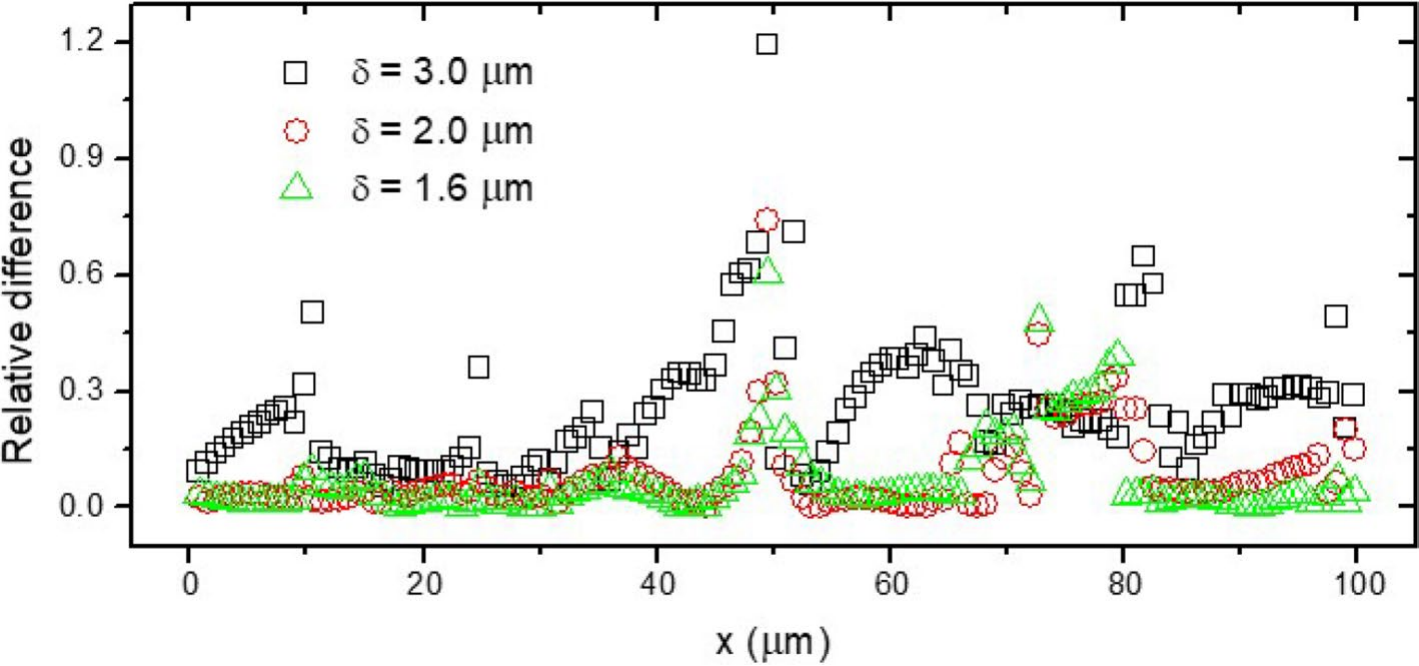
Relative differences for $\epsilon _y$ between ANSYS and three PD simulations with different horizon sizes, along the intersection of *y = 50* *μ*m and *z = 50* *μ*m planes (see top line in Fig. 8c)

To verify the peridynamic simulation results, a FE simulation is performed using ANSYS. We compare the strain concentration sites calculated by these two methods. The FE model has about 2.69 million elements, 3.66 million nodes, and equivalent boundary conditions. In the following sections, strain plots (Figs. 5, 6, 7, 8, 9, 10, 11, 12, 13 and 14) from peridynamic simulations are obtained using the 2 *μ*m horizon size (4 million nodes, see Table 2). A comparable number of nodes was used in the corresponding FE simulations shown in these figures.

As a nonlocal method, peridynamic simulations are computationally more expensive compared with FE simulations. In this work, peridynamic simulations are accelerated by using OpenACC for multicore CPUs. All simulation times listed in Table 2 are performed using 32 cores on a Linux machine with AMD Opteron 6272 CPU and 256 GB RAM. The detailed parallelization strategy is discussed in Zhang and Bobaru (2016).

As discussed in the introduction, experiments show that cracks tend to first nucleate on the free surfaces of samples under cyclic loading. We analyze the simulation results for the four free surfaces of the sample in Figs. 5 and 6. In these figures, we plot $\varepsilon _y$, the normal strain along the loading direction. All results are plotted using the same range: from *0.3%* to *0.5%*. In the FE results, the contours of grains and twins are visible.

By comparing the FE and PD simulation results, we can make the following observations:

- strain concentration sites obtained by the two models are identical except near the bottom region where the displacement boundary conditions are applied;
- while the overall strain behavior is similar, the peak strains from the peridynamic results are lower than the values obtained with the FE method. Some of these differences are caused by the different discretizations used: uniform/nonconforming grid for the PD results compared with the conforming (to the grain and twin microstructure) FE mesh;
- both FE and peridynamic results show that most strain concentrations are present at boundaries between twins, or at grain boundaries.

We also compare results in the bulk between peridynamic and FE simulations. We select the mid-planes along three axes of the sample and compare $\epsilon _y$ over these cuts. The results are shown in Figs. 7, 8, and 9, respectively. Microstructure information is provided in Figs. 7c, 8c, and 9c.

The data shows the connection between strain concentrations and twin and grain boundaries grain boundaries. As in the case of the surface analysis, the strain concentration sites in the bulk from the peridynamic simulation are identical to those from the FE analysis. Strain concentration sites near triple junctions are captured by the PD model (see Fig. 9).

Fatigue cracking in ductile materials tends to start from cyclic strain localization at certain sites as persistent slip bands appear. Because in metals the deviatoric component of the deformation is responsible for plastic deformations, we investigate how well the PD results match the von Mises strain computed by the FE model. We compare the von Mises strain values, $\epsilon _\textrm{eq}$, which is computed using the principal strain, as follows:

18 $$\begin{aligned} \epsilon _\textrm{eq} = \sqrt{\frac{2}{3} \left( (\epsilon _1 - \epsilon _2)^2 + (\epsilon _2 - \epsilon _3)^2 + (\epsilon _3 - \epsilon _1)^2 \right) } \end{aligned}$$

In Fig. 10, we show the von Mises strains from the FEM and PD solutions. Because of the loading type (along the *y*-direction), the $\epsilon _y$ (Fig. 8) and $\epsilon _\textrm{eq}$ (Fig. 10) plots are similar.

A quantitative comparison for $\epsilon _y$ strains between peridynamic and FEM results is performed along three lines on a center plane-cut (*y = 50* *μ*m, see the dotted lines in Fig. 8) of the sample. These lines are parallel to the *x*-axis, one on the front surface (*z = 50* *μ*m), another in the center (*z = 25* *μ*m), and the last on the back surface (*z = 0* *μ*m) of the sample. The PD results reported in Figs. 11, 12, and 13 are at the grid nodes, while the FE results are computed by selecting elements within a 5 *μ*m distance from the selected line and mapping (using ANSYS APDL *MOPER) results to it (recall that we use a non-uniform conforming FE mesh).

The data presented in Figs. 11, 12, and 13, shows the variation of strain across grains in the sample. The PD results with the largest horizon size (3 *μ*m, large relative to the average twin thickness) show the larger differences from the ANSYS FEM results. Some of these differences are obviously caused by the coarse, uniform grid, which does not conform to and does not approximate the microstructure well. Under a *δ*-convergence strategy, a smaller horizon is accompanied by a finer grid that gives a sharper description of the microstructure geometry. Moreover, the discussion in Strain variations across a grain boundary section mentioned the role nonlocality plays at a material interface (aligned with the uniform grid). As the horizon used becomes relatively small compared with the average grain size and average twin thickness, we notice the convergence of the PD results to the FE results. Results with the two smaller horizon sizes (2 *μ*m and 1.6 *μ*m) are similar, and the ones with 1.6 *μ*m horizon show smaller differences compared to the ANSYS results. Focusing on the peridynamic results with *δ = 1.6* *μ*m and ANSYS results along three lines, we conclude that:

- PD and FE results have similar strain variation trends. Near the sample’s free surfaces, (see Fig. 13), the surface effect in PD is still noticeable;
- Near interfaces, the nonlocality of PD solution (see Strain variations across a grain boundary section) produces smoother results compared with the sharp jump discontinuities or spikes present in the FE solution.

Point-wise relative differences between the PD and FE results for $\epsilon _y$ strains along the line considered in Fig. 11 are plotted in Fig. 14. To compute these relative differences, we first interpolate (by linear interpolation) all results to the *x*-coordinates of the grid corresponding to the $\delta = 3\,\mu$m case in the PD computations. We then obtain relative differences based on the interpolated data at these points only. The average Relative Differences, in percentages, for strain components in the loading direction, at these locations obtained with PD (for different horizon sizes) and the FEM are computed using the following formula:

19 $$\begin{aligned} \textrm{RelDiff}_{\%} = \frac{100}{n} \sum \limits _{i=1}^{n} \frac{\epsilon _\textrm{PD} - \epsilon _\textrm{FE}}{\epsilon _\textrm{FE}} \end{aligned}$$

in which *n* is number of data points where the comparisons are made. The results are listed in Table 3. The *δ*-convergence trend of the PD results to the FEM-based solution is noticed.

We observe larger differences at the locations of twin boundaries and grain boundaries. The different grids/mesh patterns used (uniform grid in PD model and conforming mesh in the FE simulations) is the major reason for the larger differences seen at interfaces. As mentioned at the beginning of this section, such differences caused by different discretizations have been reported in the FEM literature (Pyle et al. (2013)).

**Table 3:** Average relative differences in $\epsilon _y$ (along the lines shown in Fig. 8c, also used to plot the data in Figs. 11, 12, and 13) between ANSYS and three PD simulations with different horizon sizes

	$\delta = 3\,\mu \textrm{m}$	$\delta = 2\,\mu \textrm{m}$	$\delta = 1.6\,\mu \textrm{m}$
Front surface line	25.41%	8.02%	7.11%
Center section line	12.45%	7.43%	6.29%
Back surface line	26.13%	10.05%	7.43%

### The Schmid factor and nucleation of damage

Statistical studies of fatigue experiments (Krueger et al. 1992) show that cracks prefer to initiate at high maximum Schmid factor locations, favorable modulus difference (along the loading direction) and longer grain boundary between twins. The Schmid Factor (an indicator of which slip or twinning system will be active) is the ratio between the applied normal stress to the shear stress on the slip plane in the slip direction (critical resolved shear stress) (see, e.g., Gottstein (2013)). To see if there is a corelation between the locations of high maximum Schmid factors and the damage initiation locations in our PD model that simulates elastic-with-brittle-damage behavior, we compute the Schmid factors for all 12 FCC slip systems in the grains shown to be of interest in Fig. 6c (labeled as a1-b1, a2-b2, a3-b3, and a4-b4). To calculate a Schmid factor, we first compute a transformation matrix, which transforms a slip system in one grain from the grain coordinate system (CS) to the sample CS using the Euler angles of the grain. Then, the Schmid Factor is computed as $\cos (\phi )\cos (\lambda )$, where *ϕ* is the angle between the normal of the slip plane and the loading direction, and *λ* is the angle between the slip direction and the loading direction.

The Schmid factors are reported in Table 4. Two families of twin boundaries are discussed in Findley (2005): one with slip transmission (slip planes on both sides of a grain boundary intersect with it), the other one with parallel slip planes (slip planes parallel to the grain boundary). For our microstructure, the results given in Table 4 indicate the presence of transmission slips only. Modulus differences along the loading direction between four pairs of grains in Table 4 are also calculated. Two pairs, a2-b2 and a4-b4, have relatively high modulus difference (*≈ 62* GPa). The other two pairs (a1-b1, and a3-b3) have a much lower modulus difference (less than 6 GPa). Criteria proposed in Findley (2005), based on statistical studies of experimental data, indicate that cracks will nucleate at a1-b1 and a3-b3 boundaries because they have sufficiently high Schmid factors to not require a large difference in modulus for damage initiation, while the modulus difference for the a2-b2 and a4-b4 pairs is not sufficient by itself for these sites to be the first to nucleate cracks because they have lower Schmid factors. For example, a Schmid factor of 0.45 would require *≈ 150* GPa modulus difference, see Findley (2005).

**Table 4:** Schmid factor for the 12 FCC slip systems in pairs a1-b1, a2-b2, a3-b3, and a4-b4 shown in Fig. 6c. Bold values indicate maximum-by-row Schmid factors

Slip system	(−1 1 1)			(1 1 1)			(−1 −1 −1)			(1 −1 1)		
	$[0\,1\,\bar{1}]$	$[\bar{1}\,0\,1]$	$[\bar{1}\,\bar{1}\,0]$	$[0\,1\,\bar{1}]$	$[\bar{1}\,0\,1]$	$[1\,\bar{1}\,0]$	$[0\,\bar{1}\,\bar{1}]$	$[1\,0\,1]$	$[\bar{1}\,1\,0]$	$[0\,\bar{1}\,\bar{1}]$	$[\bar{1}\,0\,1]$	$[1\,1\,0]$
a1	0.0535	0.0431	0.0359	0.3124	0.1398	0.1726	0.1502	**0.4787**	0.3284	0.2157	0.3819	0.1663
b1	0.2590	0.4435	0.2829	0.2082	**0.4969**	0.2887	0.3124	0.1726	0.1398	0.1548	0.1192	0.0356
a2	0.0957	0.0473	0.0815	0.0260	0.0151	0.0109	0.0634	0.4172	0.3538	0.0582	**0.4495**	0.3913
b2	0.0784	0.0354	0.0681	0.0260	0.0151	0.0109	0.0279	0.3890	0.3611	0.0245	**0.4394**	0.4149
a3	0.4536	0.1814	0.2722	0.1194	0.2149	0.0955	**0.4870**	0.1623	0.3247	0.0859	0.2890	0.0430
b3	0.01441	0.02028	0.0058	**0.4755**	0.1057	0.3698	0.0997	0.2231	0.1232	0.3901	0.1376	0.2524
a4	0.0975	0.0483	0.0492	0.0278	0.0162	0.0116	0.0654	0.4181	0.3526	0.0600	**0.4502**	0.3902
b4	0.0783	0.0353	0.0429	0.0261	0.0151	0.0109	0.0277	0.3889	0.3611	0.0244	**0.4394**	0.4198

**Fig. 15:**
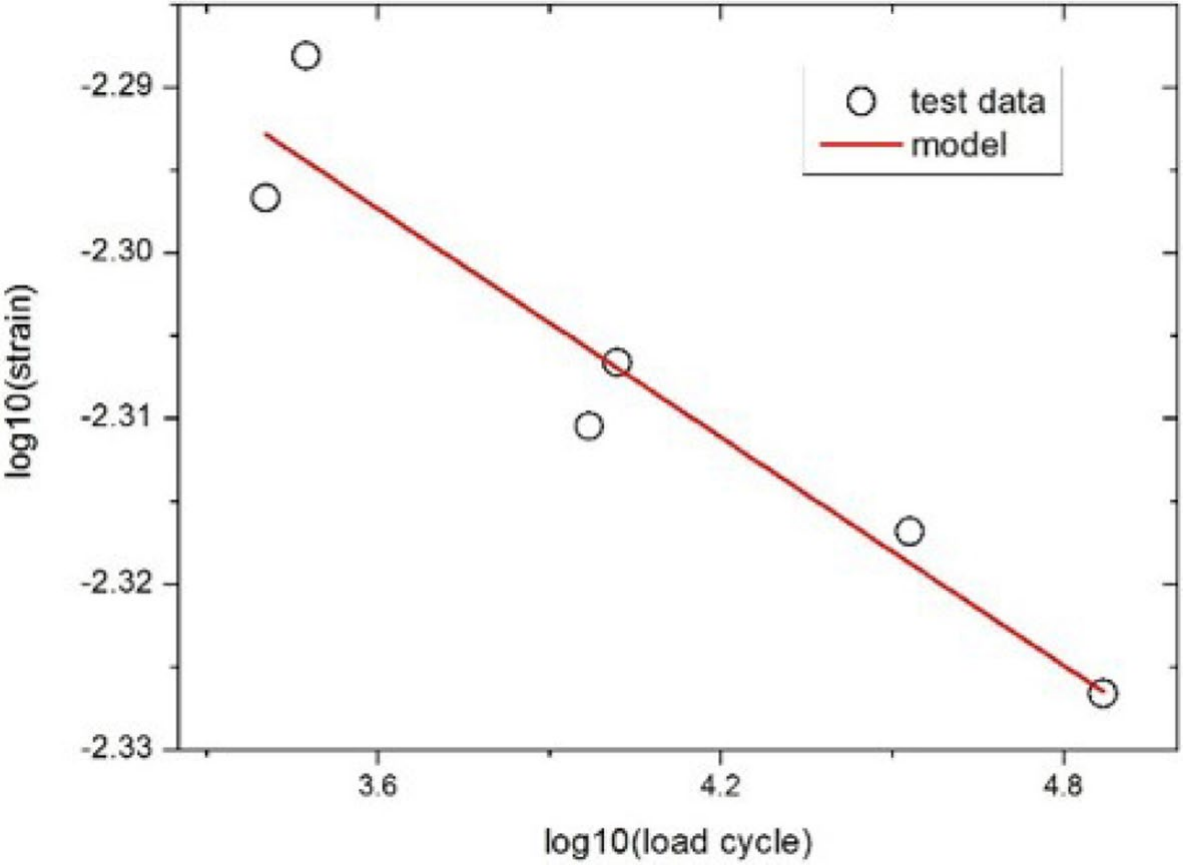
Calibration of constants in the peridynamic fatigue law for the crack nucleation phase using experimental S-N data from (Chang 2011)

Considering that a general governing law relating crack nucleation and microstructural features in polycrystalline materials is unavailable, our first attempt is to use the fatigue crack model as the one proposed in Silling and Askari (2014) to simulate crack nucleation. Implementation details and some improvements of this fatigue failure model are discussed in Zhang and Bobaru (2016); Zhang et al. (2016). Note that in the peridynamic fatigue failure model, instead of using the critical bond elongation introduced in Eqs. (7) and (9), bond breading is controlled by a new concept called remaining life. Each bond has a remaining life *λ*. The remaining life is governed by evolution laws which are calibrated to experimental data. A bond breaks if its remaining life decreases to zero. Here, we only use the first two phases of the PD fatigue fracture model, crack nucleation and propagation. Parameters in the crack nucleation are calibrated by using the S-N curve data from experiments in Chang (2011). Figure 15 shows data points from experiments in log-log plot and the linear fit used for calibration. For the crack propagation phase, we obtain parameters by enforcing a match of crack growth rate between simulation and experiments, which is fitted to Paris’ law (Findley (2005)):

20 $$\begin{aligned} \frac{da}{dN} = 4.55 \times 10^{-10} (\Delta K)^{2.45} \end{aligned}$$

The model with $2\, \mu$m horizon size is adopted in the fatigue crack simulation. To reduce the computational time, half of the original sample is used (*100 μ*m *×* *100 μ*m *×* *25 μ*m), and this model has the same back surface (see Fig. 6) as the full model considered above. This will allow us to compare the pairs of grains discussed above in relation to where damage nucleates. The loading conditions are the same as in the previous section but the applied stress magnitude is changed to 1.0 GPa in these fatigue failure simulations. In addition, a *δ /2* thickness no-fail zone is assigned at the bottom of the sample.

**Fig. 16:**
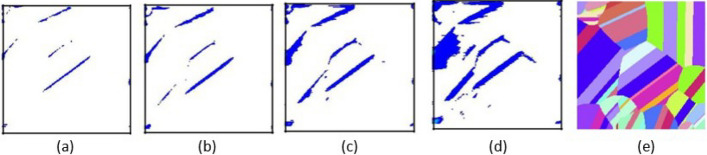
Damage maps at **a** $20\times 10^3$, **b** $40\times 10^3$, **c** $60\times 10^3$, **d** $80\times 10^3$ fatigue cycles, and **e** the corresponding microstructure of the back surface (*z = 0* *μ*m)

Figure 16 shows the evolution of nodal damage index (see Brief review of Peridynamics section) on the back surface of the sample after a number of fatigue loading cycles. The corresponding microstructure of the back surface (*z = 0* *μ*m) is also shown. A 3D rotating view of the nodal damage index in the sample at the end of the simulation is given in Video 1, in the Supplementary Materials section. The damage results show consistent patterns with the strain concentration results from Fig. 6. Notice that most nodes in the sample have a damage index smaller than 5% throughout the evolution simulated in our fatigue tests. However, on some of the boundaries, we find locations with significantly larger damage index, near 0.35 (meaning these nodes lost 35% of their bonds). These sites approach the development of a small crack (see Brief review of Peridynamics section). We can predict that cracks will develop from these sites, coalesce, and propagate along the twin grain boundary in the bulk which is already weakened by the existing damage. In addition, grain boundaries with high strain concentration present many material points with damage. These grain boundaries would be sources of cracks, as observed in experiments.

Note that the S-N and Paris curves are results of macroscale experiments. We chose to calibrate the peridynamic fatigue failure model to this data because a general governing law relating crack nucleation and microstructural features in polycrystalline materials is unavailable. The peridynamic model shows that damage nucleation locations coincide with locations where twin grain boundaries have high maximum Schmid factor. In the example shown, we found damage nucleation at the boundaries associated with all the grain pairs reported in Table 4 (see Fig. 16). However, only two of these sites satisfy the crack nucleation criteria proposed earlier in the experimental work (Stinville et al. 2017). A possible reason for this difference is that the peridynamic model used here does not include plastic deformations, which play a role in polycrystalline materials deformation. A peridynamic model enhanced with crystal plasticity capabilities should be able to capture the deformation mechanisms not covered by our current model: plastic deformations, texture evolution, etc. Another reason could be the PD surface effect. Recall (see Strain variations across a grain boundary section and Le and Bobaru (2018)) that the PD surface effect in bond-based PD introduces a surface layer, of thickness *δ*, the PD horizon, that is slightly softer and weaker than the bulk. This can influence damage nucleation when damage starts from the surface. Depending on the surface processing of an alloy, an actual surface layer with slightly different strength and elastic properties than the bulk exists. If experimental data in terms of the thickness of the actual surface layer is available, then a PD model using a horizon size in the same range as the layer thickness can be used advantageously, if that is not too small to render the calculation prohibitively costly. Future work will investigate how nucleation results change if a volume-based or energy-based PD surface correction method (Le and Bobaru (2018)) is employed.

**Remark regarding the size of the PD horizon**: reasons for selecting a horizon size smaller than the size of critical geometrical or microstructural features in the simulated sample have been discussed in the past (Bobaru and Hu (2012); Xu et al. (2018); Niazi et al. (2021)). In PD, the horizon size is the region over which the integral operator is computed, and, in some sense, this is the region over which quantities that are directly affecting the behavior at a material point are “averaged” over. One can choose to create a model that includes all fine details of the microstructure. The finer the details represented in the model, however, the higher the computational cost required. For simulating mechanical behavior of polycrystalline materials, scales from the atomistic to the grain scale can be considered. The decision which length-scale to choose comes down to balancing cost and accuracy requirements. If modeling at a certain scale cannot capture the observed behavior, finer scale models may be warranted. In the model presented here, we select the horizon size to be smaller than the average grain size to ensure that some microstructural effects are captured. The tests conducted help us answer if finer horizons sizes (with their associated higher computational costs) are justified for simulating the evolution of fatigue failure. The results shown support the conclusion that important features of failure in the polycrystalline sample are captured by using a horizon size in the range of the average grain size. While smaller horizon sizes could be used for more detailed results, the increase in cost will not be small. To reduce the computational cost, future plans include developing FCBM-PD models (Jafarzadeh et al. (2022); Mousavi et al. (2025)) for fatigue failure in alloys.

## Conclusions

A peridynamic (PD) cubic elasticity model (with brittle failure) was introduced to study strain concentration sites in a polycrystalline superalloy and their connection to brittle damage nucleation under fatigue conditions. We performed two studies for verification of the elastic response: first, we verified the jump in strain (normal to a grain boundary) against a finite element solution. Once the horizon size becomes sufficiently small (relative to the grain size), the peridynamic solution approximates well the jump discontinuity seen in the corresponding FEM solution of the classical model for the strain profile. We noted that the PD solution smooths out the strain jump-discontinuities present in the FEM solutions. This could be an advantage in modeling of real grain boundaries, which are rarely mathematically thin/sharp. We then used a synthetic model of a Ni-based superalloy with a complicated microstructure to verify the peridynamic formulation for the mesoscale problem against the corresponding classical model solved using the FEM (with ANSYS). The results obtained showed that most strain concentration locations are at twin grain boundaries. These locations were identical between the PD and FEM solutions. The convergence studies conducted showed that when the horizon size is smaller than the average microstructural features, the departures from the FEM results (in terms of normal strains) are within engineering tolerances, even if the PD solution uses uniform grids, non-conforming to the sample’s microstructure.

In contrast with the classical model, the PD model allows us to nucleate damage/cracks and have them grow and propagate with ease. We used the PD model for polycrystalline cubic elasticity endowed with a fatigue failure model to explore crack nucleation sites and the evolution of micro-damage in the polycrystalline sample under fatigue conditions. We performed a qualitative study for damage caused by fatigue loading and found that the computed damage nucleation sites correspond to places with high maximum Schmid factors. The PD model nucleated damage at more locations compared to those normally seen in experiments, likely due to the absence of plastic deformations from the model. The results obtained here using a PD horizon size in the same range as the average grain size point to the conclusion that important features of failure in the polycrystalline sample are captured by the scales used in this computational model.

The model proposed here can be used with the recent fast solvers for PD modeling of damage and failure initiation and propagation in polycrystalline materials.

## Supplementary Information

Supplementary Material 1.

## Data Availability

No datasets were generated or analysed during the current study.
